# Supplementary material for: Assessing a chip based rapid RTPCR test for SARS CoV-2 detection (TrueNat assay): A diagnostic accuracy study
Source: PLoS One. 2021 Oct 13;16(10):e0257834. doi: 10.1371/journal.pone.0257834 (PMC8513858; doi:10.1371/journal.pone.0257834)
Supplement: Attachment — Submitted filename: PLOS POINT WISE ANSWERS TO REVIEWER COMMENTS.docx [file pone.0257834.s002.docx]

**POINT WISE ANSWERS TO REVIEWER COMMENTS**

**Reviewer 1:**

1. Abbreviation of PCR should BE written at proper sections (Line no. 30, 31, 33, 35).

Answer: We have corrected the manuscript as suggested

2. Author can modify the Conclusion section of abstract.

Answer: We have modified the conclusion in abstract.

3. Advised to write the current global as well as India epidemiological data (Line no. 46, 47).

Answer: We have updated the global data and added Indian data as per your suggestion.

4. It is advised to authors to modify the sentence, as it seems duplicated from abstract section (COVID-19 testing is required before admission of a patient…..).

Answer: We have modified the sentence in revised manuscript.

5. Advised to write the updated name of RNTCP.

Ans: Name updated as National Tuberculosis Elimination Programme.

6. Author should write the abbreviation RTPCR trough out the manuscript (WHO, OPD etc).

Answer: We have incorporated this change in revised manuscript.

7. It is advised to write abbreviation in a single way (line no. 46, 47, 129, 130).

Answer: We have taken care to write abbreviation in single way in revised manuscript.

8. Write NPV and PPV in statistical analysis section also.

Answer: We have incorporated this change in revised manuscript.

9. It is advised to author that please recheck the percentage as well as sample number before writing the result.

Answer: Thank you for pointing out the typing mistake. We have corrected the data in revised manuscript.

10. Don’t use full stop sign (.) within a single sentence (Line no. 166-169).

Answer: We have incorporated this change in revised manuscript.

11. It is advised to avoid the duplication of sentences (Line no. 181- 190).

Answer: While revising the manuscript we have removed the duplicate sentences.

12. Modify the sentence (Further only 30 positive samples were tested and these reasons might have…..).

Answer: We have revised the sentence in revised manuscript.

13. Reference should be written as per the journal guideline.

Answer: Reference formatted as per Journal guidelines.

14. Reference required for actual Operational cost for TrueNat test and RTPCR.

Answer: Reference added in revised manuscript.

**Reviewer 2:**

This study is in time and address issue of quick and handy detection of the samples which can be helpful in accessing the infection rate and frequency. However, one major concern which need clarification from author.

1. Whether author compared the fidelity of this assay with the RAT (rapid antigen test), how correlative and reliable is this assay.

Answer: In this study in small subset of patients (n=30) all three test i.e. antigen detection by STANDARD Q COVID-19 Ag Test (SD Biosensor, South Korea), Truenat test and RTPCR was done. Taking RTPCR results as gold standard; Antigen detection and Truenat test showed sensitivity of 55% and 70 % respectively. The specificity in each test was 100%. Antigen detection missed 2 RTPCR positive cases (Ct value 29 and 31) but were detected by Truenat test. However, as the sample size is small this data is not included in manuscript.

2. Whether the author used simple PCR or modified nested one.

Answer: We used simple RTPCR protocol suggested by Indian council of medical research, India. All samples were tested for E gene and RdRP gene and cut off threshold <40 was considered as positive.

3. Can we identify mutated strain also in the patients.

Answer: Mutated strains of SARS Cov-2 cannot be identified by Truenat testing or routine RTPCR test.
